# Social recovery therapy in combination with early intervention services for enhancement of social recovery in patients with first-episode psychosis (SUPEREDEN3): a single-blind, randomised controlled trial

**DOI:** 10.1016/S2215-0366(17)30476-5

**Published:** 2018-01

**Authors:** David Fowler, Jo Hodgekins, Paul French, Max Marshall, Nick Freemantle, Paul McCrone, Linda Everard, Anna Lavis, Peter B Jones, Tim Amos, Swaran Singh, Vimal Sharma, Max Birchwood

**Affiliations:** a Psychology Department, University of Sussex, Brighton, UK; b Norwich Medical School, University of East Anglia, Norwich, UK; c Psychosis Research Unit, Greater Manchester Mental Health NHS Trust, Manchester, UK; d Institute of Health and Psychology, University of Liverpool, Liverpool, UK; e Lancashire Care NHS Foundation Trust, Preston, UK; f University College London, London, UK; g Institute of Psychiatry, Psychology & Neuroscience, King's College London, London, UK; h Birmingham and Solihull NHS Mental Health Foundation Trust, Birmingham, UK; i University of Birmingham, Birmingham, UK; j University of Cambridge, Cambridge, UK; k University of Bristol, Bristol, UK; l University of Warwick, Coventry, UK; m University of Chester, Chester, UK; n Cheshire and Wirral Partnership NHS Foundation Trust, Chester, UK

## Abstract

**Background:**

Provision of early intervention services has increased the rate of social recovery in patients with first-episode psychosis; however, many individuals have continuing severe and persistent problems with social functioning. We aimed to assess the efficacy of early intervention services augmented with social recovery therapy in patients with first-episode psychosis. The primary hypothesis was that social recovery therapy plus early intervention services would lead to improvements in social recovery.

**Methods:**

We did this single-blind, phase 2, randomised controlled trial (SUPEREDEN3) at four specialist early intervention services in the UK. We included participants who were aged 16–35 years, had non-affective psychosis, had been clients of early intervention services for 12–30 months, and had persistent and severe social disability, defined as engagement in less than 30 h per week of structured activity. Participants were randomly assigned (1:1), via computer-generated randomisation with permuted blocks (sizes of four to six), to receive social recovery therapy plus early intervention services or early intervention services alone. Randomisation was stratified by sex and recruitment centre (Norfolk, Birmingham, Lancashire, and Sussex). By necessity, participants were not masked to group allocation, but allocation was concealed from outcome assessors. The primary outcome was time spent in structured activity at 9 months, as measured by the Time Use Survey. Analysis was by intention to treat. This trial is registered with ISRCTN, number ISRCTN61621571.

**Findings:**

Between Oct 1, 2012, and June 20, 2014, we randomly assigned 155 participants to receive social recovery therapy plus early intervention services (n=76) or early intervention services alone (n=79); the intention-to-treat population comprised 154 patients. At 9 months, 143 (93%) participants had data for the primary outcome. Social recovery therapy plus early intervention services was associated with an increase in structured activity of 8·1 h (95% CI 2·5–13·6; p=0·0050) compared with early intervention services alone. No adverse events were deemed attributable to study therapy.

**Interpretation:**

Our findings show a clinically important benefit of enhanced social recovery on structured activity in patients with first-episode psychosis who received social recovery therapy plus early intervention services. Social recovery therapy might be useful in improving functional outcomes in people with first-episode psychosis, particularly in individuals not motivated to engage in existing psychosocial interventions targeting functioning, or who have comorbid difficulties preventing them from doing so.

**Funding:**

National Institute for Health Research.

## Introduction

Provision of early intervention services for first-episode psychosis has resulted in considerable gains in social functioning outcomes by comparison with traditional more generic mental health services.^1^, ^2^, ^3^ These services provide a range of interventions that aim to facilitate social recovery, including recovery-oriented intensive outreach case management.^4^, ^5^ Before provision of early intervention services, as few as 15% of patients would make either partial or full social recovery at 2 years;^6^ this rate increased to between 40% and 60% after provision of these services.^6^, ^7^

Nevertheless, a substantial proportion of individuals have continuing severe and persistent problems with social functioning, even after 12 months of provision of specialist early intervention services.^8^ People with poor responses to early intervention services often represent a subgroup who, although presenting with first-episode psychosis, have chronic severe and complex mental health and social functioning problems that date back premorbidly to childhood.^8^ Problems associated with social recovery in people with first-episode psychosis are complex and include poor engagement with service providers, loss of role and social contacts, perceived stigma and shame, anxiety and depression, and treatment-resistant psychotic symptoms.^9^ These persistent difficulties often result in lifelong patterns of social withdrawal.^10^ Addressing these issues at an early stage is key because the presence of persistent early social decline is associated with a poor long-term course of schizophrenia.^11^ In addition to the personal consequences of functional disability, there are also large financial implications for society, with much of the cost of psychosis resulting from lost productivity.^12^

**Research in context**

**Evidence before this study**

Problems with social functioning are common after first-episode psychosis and can persist over time, resulting in poor long-term outcomes. We searched PubMed from inception to July 19, 2017, with the search terms “first episode psychosis”, “social functioning”, and “psychological treatments”. We identified 19 papers, of which ten were relevant because they referred to factors influencing recovery from psychosis. However, few studies assessed interventions targeting social recovery as a primary outcome. Meta-analyses of psychological interventions for psychosis have shown some evidence for the effectiveness of cognitive behavioural therapy on functioning as a secondary outcome, but its primary focus is on reducing the positive symptoms of psychosis. Other psychosocial interventions, including individual placement and support, cognitive remediation, and social skills training, all target functional outcomes, but each focuses on specific aspects of functioning, such as return to work and skills acquisition. Further research is needed focusing on a wider construct of social recovery and including individuals who might find it difficult to engage in existing psychosocial interventions because of comorbid difficulties.

**Added value of this study**

Our findings show that social recovery therapy plus early intervention services is superior to early intervention services alone on the primary outcome of time spent in structured activity. Individuals who participated in this study had complex difficulties warranting intensive and targeted intervention. Participants were young people with extreme social withdrawal who also had a wide range of other complex comorbidities, including high levels of treatment-resistant and residual positive psychotic symptoms, negative symptoms, and anxiety and depression, as well as the presence of current hopelessness and a lack of hope for the future. To our knowledge, this is the first study to show a significant improvement in functioning in an already highly disabled group at a crucial stage in life.

**Implications of all the available evidence**

Social recovery therapy could be useful in improving functional outcomes in people with first-episode psychosis, particularly in individuals not motivated to engage in existing psychosocial interventions targeting functioning, or who have comorbid difficulties that prevent them from doing so.

New interventions targeting functional and social recovery are needed in people with first-episode psychosis. Conventional cognitive behavioural therapy (CBT) for psychosis has shown some evidence of effectiveness on social disability as a secondary outcome, even when the primary focus has been on reducing positive symptoms of psychosis.^13^ More specific adaptations of CBT for psychosis targeting negative symptoms and social recovery have shown promise.^14^, ^15^, ^16^ Functional interventions such as individual placement and support have been effective in helping individuals to return to paid employment.^17^ However, this intervention is most successful in individuals who are motivated to engage and wish to work, and might be less effective in individuals who have poor engagement, are ambivalent about change, and continue to have comorbid difficulties.^17^ Intervention needs to target a wider construct of social recovery than work alone, such as education and voluntary work, and household activity and child care, which are productive economic activities. Prosocial activity with peers is also crucial in ensuring that young people achieve development milestones and continue to thrive at a key stage in life. Such principles are consistent with the user-oriented goals identified by the recovery movement.^18^, ^19^

We have developed an intervention that focuses on social recovery. The rationale behind social recovery therapy^9^ is that in-vivo multisystemic assertive outreach and case management are necessary to encourage socially withdrawn individuals back into social environments, whereas the techniques of CBT are necessary to promote engagement and overcome the symptoms that impede this. The intensive and novel combination of these elements of therapy provide the possibility of making meaningful changes to the lives of very withdrawn and difficult to engage young people who have not previously responded to standard early intervention services provision.

Preliminary evidence for the efficacy of social recovery therapy derives from the ISREP MRC Trial Platform Study,^14^ which suggested that the intervention can improve social recovery in individuals in the early stages of psychosis and is cost-effective.^20^ In that trial, unemployed adults with up to 8 years of history of non-affective psychosis showed clinically and statistically significant improvements in structured activity, symptoms, hopelessness, and rates of employment after 9 months of social recovery therapy. Although these findings are promising, targeting social disability at an even earlier stage might improve outcomes.

We did the SUPEREDEN3 trial to assess the efficacy of adding social recovery therapy to early intervention services, with the aim of achieving a step-change in early social recovery in young people who have severe and persistent social disability despite receiving early intervention services for more than 1 year after their first episode of psychosis. The primary hypothesis was that augmentation of early intervention services with social recovery therapy would lead to improvements in time spent in structured activity at 9 months. Secondary hypotheses were that the effects on activity would persist at 15 months and that there would be benefits on general psychopathology and negative symptoms.

## Methods

### Study design and participants

We did this single-blind, phase 2, randomised controlled trial at four well established early intervention services in the UK. Ethics approval was granted by the National Research Ethics Service Committee in the Black Country, West Midlands (reference 12/WM/0097). We did the trial in accordance with the Good Clinical Practice guideline.

Eligible patients were aged 16–35 years; had non-affective psychosis; were clients of early intervention services in Birmingham, Lancashire, Norfolk, and Sussex (for entry into early intervention services in the UK, individuals are required to have psychotic symptoms with a Positive and Negative Syndrome Scale [PANSS] score of ≥4); had been clients of early intervention services for 12–30 months; and had low levels of structured activity after at least 1 year of treatment in early intervention services (defined as ≤30 h/week on the Time Use Survey^21^). We excluded patients if they were part of the original National EDEN cohort,^22^ did not speak adequate English to engage in the intervention, and were deemed too unwell to engage with the intervention. Potential participants were approached by their care coordinator and asked if they were willing to discuss the trial with a research assistant. Information about the trial was shared verbally and via the participant information sheet.

All participants provided written informed consent. Participants were made aware that they could withdraw at any time, without any consequences for their treatment.

### Randomisation and masking

Participants were randomly assigned (1:1), via an automated, concealed, computer-generated allocation sequence generated by Norwich Clinical Trials Unit, to receive social recovery therapy plus early intervention services (intervention) or early intervention services alone (control). Randomisation was done in permuted blocks (sizes of four to six) and was stratified by sex and recruitment centre (Norfolk, Birmingham, Lancashire, and Sussex).

By necessity, participants were not masked to group allocation, but allocation was concealed from outcome assessors. An email notification of the allocation was sent automatically to therapists and the trial manager. An email notification confirming that the participant had been randomly assigned (with no information about group allocation) was sent to the research assistant, thus keeping the trial team masked to group assignment. Therapists were also required to consider potential breaches in masking, and participants were reminded by assessors not to disclose treatment allocation. When masking was broken, another rater who was masked to group assignment assessed the participant at all subsequent timepoints.

### Procedures

All participants received provision of early intervention services from specialist teams. The centres in Birmingham, Lancashire, Norfolk, and Sussex are all recognised centres of excellence for delivery of early intervention services. High fidelity to the early intervention services model indicated that the centres had the ability to deliver a comprehensive range of interventions.^22^ These interventions include intensive and assertive recovery-oriented case management, supported employment, peer support, group interventions, family work and CBT for psychosis, and psychiatric medications and medical and psychiatric monitoring. All participants had an early intervention services case manager who provided oversight of their care and remained in contact with the participant throughout the trial.

In the intervention group, early intervention services were augmented by social recovery therapy delivered by a therapist who was trained and supervised by the trial team. All therapists were supervised and accredited CBT therapists with experience in the relevant early intervention services. Social recovery therapy in this trial was designed and supervised to ensure it was done in partnership with the early intervention services.

DF, PF, and JH developed the specific therapeutic procedures used in social recovery therapy. These procedures draw from our experience in the ISREP MRC Trial Platform^14^ and details are provided in our published manual.^9^ In brief, social recovery therapy is delivered in three stages.

Stage one involves engagement and development of a formulation, which comprises establishment of a working therapeutic relationship to facilitate engagement and identify a problem list. Alongside this approach is a detailed assessment of personal motivation and premorbid hopes, expectations, and goals, which might have changed with respect to the effect of illness. Specific behavioural assessment is done in vivo to assess how symptoms affect activity. Links are identified between personally meaningful values and goals and achievable day-to-day activity targets. Stage two involves preparing for new activities, whereby the client and therapist work together to identify pathways to meaningful new activities. This strategy includes referral to relevant vocational agencies, education providers, and community providers of social or sports activities. Cognitive work at this stage involves promoting a sense of agency and addressing hopelessness, feelings of stigma, and negative beliefs about self and others. Behavioural experiments start focusing on managing symptoms while engaging in activity. Stage three involves engagement in new activities, which involves the active promotion of social activity using behavioural experiments, and fostering feelings of mastery and agency. The behavioural experiments are progressively shaped to address specific problems presented by individuals. Therapists adopt an assertive outreach style of contact, most frequently visiting people at home or in community settings. Therapists are also encouraged to work systematically with family members, employers, and educational providers to discuss and overcome potential problems that could impede social recovery.

All trial therapists had formal training in CBT. Additionally, therapists received specialist training in the social recovery therapy approach via workshops and regular supervision sessions from the trial therapy team, including DF, PF, and JH. The Cognitive Therapy Scale-Revised (CTS-R)^23^ was used to ensure therapist competence. All therapists were required to score more than 36 from tape-rated sessions and an average of more than 3 on each item. Adherence to the social recovery therapy model was also assessed with a specific checklist. This method used a combination of independent expert rating of case notes and therapist ratings of individual sessions to assess techniques applied in individual sessions. A sufficient dose of therapy was defined as at least six sessions, including the presence of an assessment and formulation phase and active behavioural experiments occurring in at least two independent sessions.

### Outcomes

Study assessments took place at baseline, 9 months (post-intervention), and 15 months (6 months' follow-up). Research assistants visited participants at home to undertake assessments and used flexible strategies to maintain engagement with participants, such as rearranging missed appointments to maintain participation. Inter-rater reliability on outcome measures was ensured via regular training sessions for research assistants and fortnightly telephone supervision meetings to discuss assessment queries.

The primary outcome was structured activity at 9 months, as measured by the Time Use Survey adapted for work in this client group.^21^ The Time Use Survey is a semi-structured interview that enquires about time spent over the past month on work, education, voluntary work, leisure, sports, housework or chores, and child care. Time spent on each of the activities is calculated in terms of the average number of hours per week. The activities are summed to create two scores: constructive economic activity (work, education, voluntary work, housework or chores, and child care) and structured activity (constructive economic activity plus leisure and sports activities). We adapted the Time Use Survey from a version developed by the UK Office for National Statistics,^24^ enabling activity levels to be directly compared with age-matched non-clinical peers. On average, a non-clinical group aged 16–36 years engage in 63·49 h of structured activity per week, and activity levels below 30 h are indicative of poor social functioning.^21^ The intraclass correlation coefficient for inter-rater reliability on the Time Use survey was 0·99.^24^ The Time Use Survey was also administered at 15 months. Structured activity and constructive economic activity at 15 months were recorded to explore the tenacity of the treatment effect; however, the statistical analysis plan is clear that the primary outcome for the study was the Time Use Survey at 9 months.

We used the PANSS^25^ to assess general psychopathology and negative symptoms as secondary outcomes. Other prespecified secondary outcomes at months 9 and 15 were the Schedule for the Assessment of Negative Symptoms,^26^ the Social Interaction Anxiety Scale (SIAS),^27^ the Beck Depression Inventory-II,^28^ the Beck Hopelessness Scale (BHS),^29^ the Meaning in Life Questionnaire (MLQ),^30^ and the Adult Trait Hope Scale.^31^

The Client Service Receipt Inventory^32^ and the EuroQol-5D^33^ were included as secondary outcomes in the trial protocol as part of a health economic evaluation of the intervention; results will be reported elsewhere.

A range of other scales were included to assess potential mediation of outcome. The Quality of Life Scale^34^ and the Role and Social Global Functioning Scales^35^ were included in the original trial protocol as secondary outcomes, but will be reported in another paper investigating mediation effects. Other mediator variables included the Schizotypal Symptoms Inventory,^36^ the Brief Core Schema Scales,^37^ and a range of neuropsychological variables. Data for these variables are also the subject of another paper investigating mediation effects.

Serious and adverse events were recorded over the course of the trial by use of standard operating procedures and reported to the National Health Service research ethics committee.

### Statistical analysis

Data were collected according to the original funded protocol as submitted to and approved by the National Research Ethics Service Committee. A briefer protocol summarising only the primary and secondary outcomes was published as the registered trial protocol. We report all data collected in this paper.

Data were analysed according to a statistical analysis plan developed before unmasking of the data on Nov 6, 2015. The statistical analysis plan differed from the original ISCRTN trial protocol in that it was more specific in identifying the analysis of primary outcome as time use at 9 months, and also identified a range of secondary outcomes. These changes to the analysis plan took place after publication of the ISCTRN protocol in June, 2012, after further methodological review and taking into account considerations of multiplicity for the analysis of primary outcome, in particular avoiding type 1 error through multiplicity.^38^ These changes were discussed with and approved by the trial steering committee, and were recorded in the trial documentation.

The planned recruitment to the trial was based on a sample size of 150 individuals. A consensus group of clinicians and service users had conservatively estimated the minimum clinically significant gain on the primary outcome as 4 h on the Time Use Survey. With a standard deviation of 8 h and a minimum clinically important difference of 4 h, the study would have 90% power to detect a difference of 4 h to be significant at the conventional (two-sided) α level of 5%. The trial as designed had 80% power to find the same effect with 120 evaluable patients.

The primary outcome was assessed with generalised mixed models, with an identity link and Gaussian mixed error. Each participant contributed two observations: one at baseline and one at 9 months. The model included indicators for whether the observation was at baseline or 9 months, and whether or not the participant was randomly assigned in that period to receive the active intervention or control strategy. All models included the stratification variables as patient-level explanatory factors (sex and recruitment centre). Observations were linked with random intercept terms within a patient. Denominator degrees of freedom were derived from the number of patients. Supportive analyses were done with the separate components of the primary outcome. The primary outcome was analysed with residual (r-side) random effects rather than generalised (g-side) random effects. We did a further supportive analysis with baseline score as a patient-level explanatory variable.

We did the principal analysis in all available participants by intention to treat, with no imputation of missing values. Missing data were assumed to be missing not at random. Supportive analyses addressed missing data patterns by modelling jointly the continuous outcome score (with Gaussian error) and observed loss to follow-up (with Bernoulli error) to describe the joint probability of the observed outcome. The joint (multivariate) models required the assumption that individuals with missing data have, on average, lower social functioning than those who do not drop out. This defensible assumption simply implies that loss to follow-up is a measure of lower scores on the scale of interest.

All analyses were done with SAS (version 9.4). This trial is registered with ISRCTN, number ISRCTN61621571.

### Role of the funding source

The funder of the study had no role in study design, data collection, data analysis, data interpretation, or writing of the report. The corresponding author had full access to all the data in the study and had final responsibility for the decision to submit for publication.

## Results

Between Oct 1, 2012, and June 20, 2014, we randomly assigned 155 participants (n=39 from Birmingham, n=53 from Lancashire, n=47 Norfolk, and n=16 Sussex) to receive social recovery therapy plus early intervention services (n=76) or early intervention services alone (n=79); the final sample comprised 154 patients after one participant withdrew and requested that their data be removed (figure).

**Figure:**
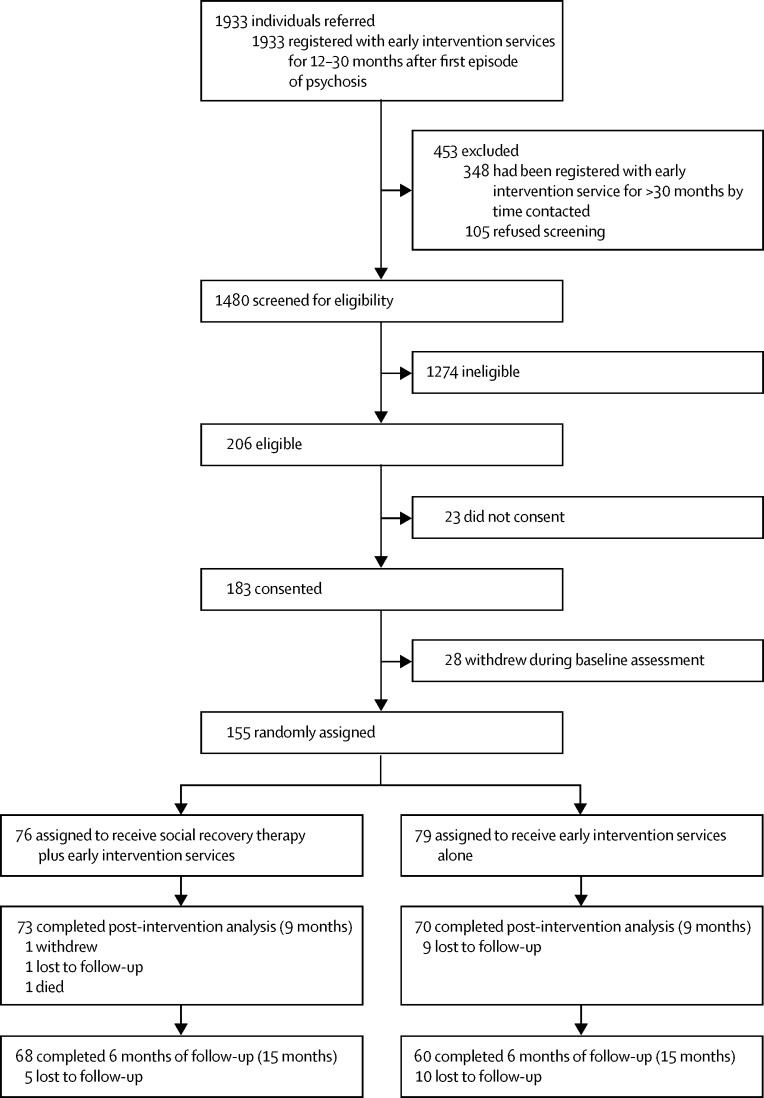
Trial profile

Baseline characteristics were similar between groups (table 1). Participants were predominantly men, single, and of White British ethnicity (table 1). Length of illness and duration of untreated psychosis were variable, but, as per the study inclusion criteria, all participants had been engaged with early intervention services for 12–30 months (table 1). Of note is the severity of social disability, psychotic symptomatology, anxiety, and depression present in the sample at baseline (table 2).

**Table 1:** Baseline characteristics

		**Early intervention services alone (n=79)**	Social recovery therapy plus early intervention services (n=75)*
Sex			
	Female	19 (24%)	19 (25%)
	Male	60 (76%)	56 (75%)
Age (years)		24·15 (22·17–27·79)	24·84 (20·73–29·04)
Length of illness (months)		26 (20–38)	23·5 (17–33)
Duration of untreated psychosis (days)		66 (20–240·75)	73 (13–316)
Premorbid adjustment score			
	Early adolescence	0·34 (0·15)	0·33 (0·20)
	Late adolescence	0·36 (0·17)	0·34 (0·17)
Time in schooling (years)		12 (11–12)	12 (11–13)
Ethnic group			
	British	58 (73%)	55 (73%)
	Irish	2 (3%)	1 (1%)
	Any other white background	1 (1%)	2 (3%)
	White and black Caribbean	2 (3%)	0
	White and black African	0	1 (1%)
	White and Asian	0	2 (3%)
	Any other mixed background	0	1 (1%)
	Indian	1 (1%)	0
	Pakistani	6 (8%)	7 (9%)
	Bangladeshi	1 (1%)	1 (1%)
	Any other Asian background	1 (1%)	1 (1%)
	Black Caribbean	4 (5%)	0
	Black African	2 (3%)	1 (1%)
	Any other black background	0	1 (1%)
	Any other ethnic group	1 (1%)	2 (3%)
Mother tongue			
	English language	75 (95%)	69 (92%)
	Other language (but with a good knowledge of English language)	4 (5%)	6 (8%)
Marital status			
	Single	69 (87%)	67 (89%)
	Cohabiting	5 (6%)	4 (5%)
	Married	3 (4%)	4 (5%)
	Divorced	2 (3%)	0

Data are n (%), median (IQR), or mean (SD), unless otherwise specified.

* Minus one participant who withdrew and requested that their data be removed.

**Table 2:** Primary and secondary outcomes

	**Baseline**		**9 months**		**15 months**	
	Early intervention services alone	Social recovery therapy plus early intervention services	Early intervention services alone	Social recovery therapy plus early intervention services	Early intervention services alone	Social recovery therapy plus early intervention services
**Primary outcomes**						
Structured activity	12 (8·6); n=79	11 (7·5); n=75	18 (20); n=70	26·6 (24·2); n=73	22·5 (23·3); n=60	23 (19); n=68
Constructive economic activity	7·9 (7·5); n=79	7·5 (6·1); n=75	14·1 (20); n=70	20·1 (22); n=73	16·5 (23·3); n=60	16·4 (17); n=68
**Secondary outcomes**						
PANSS	65 (49–76); n=79	62 (40–73); n=75	58 (45–73); n=57	54 (39–73); n=66	56 (40–65); n=46	48 (39–67); n=57
SANS	27 (18–36); n=78	23 (14–35); n=74	18 (11–34); n=57	19 (8–23); n=64	21 (9–30); n=47	20 (7–33); n=57
BDI	19 (8–29); n=75	18 (8–27); n=73	16 (7–23); n=55	12 (4–25); n=62	8 (4–20); n=43	9 (4–22); n=55
SIAS	40 (30–52); n=72	40 (31–51); n=69	37 (30–50); n=53	37 (22–50); n=64	34 (17–47); n=43	36 (24–51); n=56
BHS	9 (6–13); n=67	8 (4–12); n=67	7 (4–13); n=56	5 (3–10); n=59	5 (3–11); n= 42	4 (2–8); n=56
ATHS sense of agency	18 (11–22); n=67	15 (10–20); n=68	17 (11–23); n=46	19 (12–23); n=54	19 (14–24); n=40	21 (17–24); n=53
ATHS optimism	19 (13–24); n=67	20 (15–23); n=68	20 (16–24); n=46	21 (16–23); n=54	20 (17–23); n=40	22 (18–25); n=53
MLQ	42 (34–48); n=68	39 (31–46); n=67	42 (37–49); n=46	40 (35–48); n=56	41 (34–48); n= 40	40 (34–46); n=52

Data are mean (SD) or median (IQR). PANSS=Positive and Negative Symptom Scales. SANS=Scale for the Assessment of Negative Symptoms. BDI=Beck Depression Inventory. SIAS=Social Interaction Anxiety Scale. BHS=Beck Hopelessness Scale. ATHS=Adult Trait Hope Scale. MLQ=Meaning in Life Questionnaire.

Participants allocated to receive social recovery therapy plus early intervention services received a mean of 16·49 sessions of social recovery therapy (SD 8·39; range 0–37). Competence in cognitive therapy as assessed by the CTS-R was excellent: of the random selection of therapy tapes rated independently, 27 (90%) of 30 scored higher than the cutoff based on the therapy protocol. Adherence ratings on the social recovery CBT checklist indicated that 61 (81%) participants received a sufficient dose of social recovery therapy. Seven (9%) participants dropped out and did not have ratings available, and seven (9%) did not receive a sufficient dose.

143 (93%) participants had data for the primary outcome at 9 months (n=12 [7%] had missing data overall: n=3 in the social recovery therapy plus early intervention services group and n=9 in the early intervention services alone group; figure, table 2). The primary intention-to-treat analysis showed that social recovery therapy plus early intervention services was associated with a large and clinically important increase in structured activity of 8·1 h (95% CI 2·5–13·7; p=0·0050) compared with early intervention services alone (table 3). The supportive analysis using a repeated measure analysis (residual random effect) provided a similar estimate (8·8 h, 95% CI 1·3–16·3; p=0·021). The supportive analysis using baseline value as a patient-level explanatory variable provided an estimate of 8·9 h (95% CI 1·4–16·5; p=0·021).

**Table 3:** Prespecified outcome analysis and joint models for primary and secondary outcomes

	**Effect size (95% CI)**	p value (intervention *vs* control)*	**Missing data**		**p value**‡
			Early intervention services alone (n=79)	Social recovery therapy plus early intervention services (n=75)†	
**Primary outcomes**					
Structured activity at 9 months	8·080 (2·502 to 13·657)	0·0050	9 (11%)	2 (3%)	0·011
Constructive economic activity at 9 months	5·859 (0·790 to 10·928)	0·024	9 (11%)	2 (3%)	0·034
**Secondary outcomes**					
Structured activity at 15 months	0·054 (−5·154 to 5·262)	0·98	19 (24%)	7 (9%)	0·037
Constructive economic activity at 15 months	−0·506 (−5·048 to 4·036)	0·83	19 (24%)	7 (9%)	0·046
Positive PANSS 9 months	0·306 (−1·228 to 1·840)	0·69	22 (28%)	9 (12%)	0·068
Negative PANSS 9 months	−1·020 (−2·662 to 0·622)	0·22	22 (28%)	9 (12%)	0·032
General PANSS 9 months	−1·014 (−3·514 to 1·486)	0·42	22 (28%)	9 (12%)	0·043
Positive PANSS 15 months	1·219 (−0·632 to 3·071)	0·19	32 (41%)	18 (24%)	0·071
Negative PANSS 15 months	−0·629 (−2·411 to 1·152)	0·49	32 (41%)	18 (24%)	0·073
General PANSS 15 months	−0·084 (−3·031 to 2·862)	0·96	33 (42%)	18 (24%)	0·081
SANS total at 9 months	9·713 (−14·568 to 33·994)	0·43	20 (25%)	11 (15%)	0·17
SANS total at 15 months	16·798 (−10·553 to 44·147)	0·23	32 (41%)	18 (24%)	0·035
BDI at 9 months	−1·567 (−4·840 to 1·706)	0·35	24 (30%)	13 (17%)	0·10
BDI at 15 months	0·748 (−3·261 to 4·757)	0·71	36 (46%)	20 (27%)	0·067
SIAS at 9 months	−2·559 (−6·964 to 1·846)	0·25	26 (33%)	11 (15%)	0·016
SIAS at 15 months	1·490 (−4·132 to 7·111)	0·60	36 (46%)	19 (25%)	0·10
BHS at 9 months	−1·464 (−3·282 to 0·354)	0·11	33 (42%)	16 (21%)	0·020
BHS at 15 months	−1·451 (−3·257 to 0·355)	0·11	37 (47%)	19 (25%)	0·022
ATHS total score 9 months	2·214 (−1·504 to 5·931)	0·24	33 (42%)	21 (28%)	0·15
ATHS total score 15 months	3·860 (−0·266 to 7·987)	0·066	39 (49%)	22 (29%)	0·0060
MLQ total score 9 months	2·193 (−1·496 to 5·883)	0·24	33 (42%)	19 (25%)	0·12
MLQ total score 15 months	0·782 (−3·196 to 4·759)	0·70	39 (49%)	23 (31%)	0·043

Data are n (%), unless otherwise specified. This approach assumes that loss to follow-up is associated with poor performance on the scale of interest. PANSS=Positive and Negative Symptom Scales. SANS=Scale for Assessment of Negative Symptoms. BDI=Beck Depression Inventory. SIAS=Social Interaction Anxiety Scale. BHS=Beck Hopelessness Scale. ATHS=Adult Trait Hope Scale. MLQ=Meaning in Life Questionnaire.

* p value from completer case analysis.

† Minus one participant who withdrew and requested that their data be removed.

‡ p value from joint modelling (multivariate) analyses done to account for missing data.

Despite considerable effort to retain participants, more data were missing for secondary outcomes than for primary outcomes, particularly for face-to-face assessments (table 3). For time use at 15 months, data were missing for 26 (16%) participants overall; PANSS data were missing for 31 (20%) participants at 9 months and 50 (35%) participants at 15 months (table 3). The pattern of missing data was biased, with more data missing in the control group than the intervention group (table 1), and was thus regarded as missing not at random.

Completer case analysis of treatment effects for time use and other secondary outcomes at 15 months showed no systematic differences between experimental conditions (table 3). However, results of the joint models provided supportive evidence that, conditional on the assumption that loss to follow-up is associated with a poorer score on time use, the observed results are consistent with systematic differences in several secondary outcomes, including structured and constructive economic time use at 15 months, PANSS negative symptoms and general psychopathology at 9 months, SIAS at 9 months, BHS at 9 months and 15 months, Trait Hope at 15 months, and MLQ at 15 months (table 3).

The independent data monitoring and ethics committee recorded no adverse or serious adverse events attributable to study therapy.

## Discussion

The main aim of this study was to establish the presence of a treatment effect in a subgroup of patients who are known to be hard to treat, tend not to engage and have complex problems, and are the poorest outcome early intervention subgroup for whom no effective treatment currently exists. The study was powered to detect an effect on the primary outcome at 9 months; the result at that timepoint is clear and definitive. Participants who received social recovery therapy plus early intervention services had a large, significant, and clinically important improvement of greater than 8 h per week in their level of structured activity compared with those receiving early intervention services alone. To our knowledge, this is the first study to show a significant improvement in functioning in this already highly disabled group. The size of the effect is twice that identified by consensus groups of users and clinicians as the minimum clinically important difference, and represents an amount of activity equivalent to a working day. Evidence from the primary analysis is therefore clear that social recovery therapy might have a clinically important effect in promoting earlier social recovery by comparison with early intervention services alone.

The key issue for secondary outcomes was whether or not this effect persisted at 15 months. The main problem was a high rate of missing data at that point. This level of missingness meant that all analyses of secondary outcomes had low statistical power and were difficult to interpret. This population is incredibly challenging to retain in follow-up and the excellent response rate for the primary outcome is attributable to the efforts of the field researchers involved in the study. Missingness is clearly related to the outcomes of interest because availability for assessments by participants is related to social engagement, and social recovery therapy specifically aims to increase social engagement. At all assessment points many more participants were available for follow-up assessments in the social recovery therapy group than in the early intervention services alone group. At 15 months, the proportion of participants lost to follow-up for structured activity was more than two-times higher in the control group than in the intervention group. This difference in itself might imply an effect of the intervention on engagement that most clinicians would regard as useful and worthy of examination in a future study. However, the pattern of differential missingness represents a challenge in interpretation of the effect at 15 months because it might formally be regarded as missing not at random.

Results from completer analysis suggest that outcomes at 15 months were similar between the groups, but this finding might be biased if participants who dropped out were those with the worst outcomes. The joint models analysed simultaneously the outcome of interest and the binomial of missing values for that outcome, and were intended to provide a least biased assessment appropriate to the data being missing not at random. Modelling of the two values simultaneously in the multivariate model accounts for bias and provided a more encouraging overall p value for the difference between the two study groups than that from the principal analysis. Although reliant on the assumption that dropout equals worse outcome on the scale of interest, the joint modelling analyses provide encouragement that at least some of the effect of the experimental treatment on time use could remain at 15 months, albeit with a level of attenuation when compared with the similarly derived 9 month values. A limitation of the joint modelling approach is it provides a p value only for the combined pseudolikelihoods of the outcome and dropout, and cannot provide an updated estimate or confidence interval for the treatment effect.

A further question regarding the secondary outcomes was the effect on negative symptoms and general psychopathology as assessed by PANSS. Again, a high level of missing data does not allow a firm conclusion to be made about these outcomes and the completer analysis suggests there is no effect, but the joint modelling, which attempts to account for missing data, suggests an effect on these outcomes at 9 months.

Of the individuals identified as meeting inclusion criteria, three-quarters consented and were randomised into the study. This level of recruitment was the result of intensive and assertive procedures. The research assistants worked in an assertive outreach manner, visiting participants at home and engaging them into the study. The result was the recruitment of a group with very severe and stable social disability. The total time spent in activity by the group recruited for the study was less than 12 h per week, compared with more than 60 h per week in an age-matched non-clinical sample.^21^ This non-clinical sample comprised a group of young people with extreme social withdrawal who also had a wide range of other complex comorbidities, including high levels of treatment-resistant and residual positive psychotic symptoms, negative symptoms, anxiety and depression, as well as presence of current hopelessness and a lack of hope for the future.

The present findings extend those from the ISREP MRC Trial Platform Study, which compared the effectiveness of social recovery therapy versus treatment as usual and reported benefits at 9 months, 15 months, and 2 year follow-up, and associated gains in secondary outcomes and cost-effectiveness.^14^, ^20^ Our study represents a more rigorous test in view of the comparator treatment-as-usual intervention of early intervention services—a highly active treatment that consisted of high-quality provision of early intervention services in sites with good fidelity to the early intervention services model, as recommended by guidelines from the National Institute for Health and Care Excellence for psychosis and schizophrenia.^39^ Improvements were observed in both treatment groups, but with differential improvement in the social recovery therapy group.

A strength of this study was good internal and external validity of the trial design for the primary outcome. The study was done with a high degree of rigour, with all researchers and therapists involved in the study receiving regular supervision and with routine checks on inter-rater reliability and adherence to the therapy model.

A limitation of the study was that it was compromised by the level and pattern of missing data in the secondary outcomes, albeit one that we addressed by use of joint modelling. The characteristics of the target group in this study, as a difficult to engage and extremely withdrawn sample, represent a challenge to researchers, especially when follow-up assessments are reliant on face-to-face communications. Future studies in this area might maximise rate of follow-up by focusing on hard proxy variables of engagement in services that could be derived from records rather than face-to-face assessments, especially as engagement is of itself an important outcome in this population. A further limitation of this study was that many of the secondary outcomes had wide confidence intervals (an indication of low statistical power), suggesting low precision or uncertainty in the estimation of treatment effects. Furthermore, the study was by necessity single blind, and thus could be affected by the experience of the participant in receiving therapy.

The target population for this study was a particularly high-severity group of young people with first-episode psychosis who are likely to have poor long-term outcomes. This study provides encouragement for practitioners in early intervention services to focus on this subgroup who are often neglected. Our results also suggest that social recovery therapy techniques could be a useful addition in this group. This is the first study to show benefits in this population. The effect size after treatment is clearly of clinical benefit, especially given the extreme social withdrawal present at baseline. Furthermore, the differential dropout rate between groups, with many more participants available for follow-up in the treatment group than in the control group, implies benefits on maintenance of engagement in the treatment group, which could be important to assess as an outcome in future work. The degree to which the treatment results in persistence of gains and longer term effects likewise deserves further study. Joint modelling aimed to account for the bias associated with the differential pattern of missingness in the analysis and suggests the potential for persistence of effects in the long term, but because this was a supplementary analysis, persistence of gains cannot be established. A larger more definitive trial is required to examine long-term effects more clearly. The extent to which interventions of this type might be enhanced by top-up therapy or a greater number of sessions over a longer duration could be worthy of further scrutiny in view of evidence from similar studies in adults with more established schizophrenia.^16^
